# RNA Sequencing Revealed Numerous Polyketide Synthase Genes in the Harmful Dinoflagellate *Karenia mikimotoi*

**DOI:** 10.1371/journal.pone.0142731

**Published:** 2015-11-11

**Authors:** Kei Kimura, Shujiro Okuda, Kei Nakayama, Tomoyuki Shikata, Fumio Takahashi, Haruo Yamaguchi, Setsuko Skamoto, Mineo Yamaguchi, Yuji Tomaru

**Affiliations:** 1 National Research Institute of Fisheries and Environment of Inland Sea, Fisheries Research Agency, 2-17-5 Maruishi, Hatsukaichi, Hiroshima, Japan; 2 Research Fellow of the Japan Society for the Promotion of Science, Kojimachi Business Center Building, 5-3-1 Kojimachi, Chiyoda-ku, Tokyo, Japan; 3 Niigata University Graduate School of Medical and Dental Sciences, 1–757 Asahimachi-dori, Chuo-ku, Niigata, Japan; 4 Center for Marine Environmental Studies (CMES), Ehime University, 2–5 Bunkyo-cho, Matsuyama, Ehime, Japan; 5 Department of Biotechnology, College of Life Sciences, Rhitsumeikan University, 1-1-1 Noji-higashi, Kusatsu, Shiga, Japan; 6 Laboratory of Aquatic Environmental Science, Faculty of Agriculture, Kochi University, 200, Monobe, Nankoku, Kochi, Japan; King Abdullah University of Science and Technology, SAUDI ARABIA

## Abstract

The dinoflagellate *Karenia mikimotoi* forms blooms in the coastal waters of temperate regions and occasionally causes massive fish and invertebrate mortality. This study aimed to elucidate the toxic effect of *K*. *mikimotoi* on marine organisms by using the genomics approach; RNA-sequence libraries were constructed, and data were analyzed to identify toxin-related genes. Next-generation sequencing produced 153,406 transcript contigs from the axenic culture of *K*. *mikimotoi*. BLASTX analysis against all assembled contigs revealed that 208 contigs were polyketide synthase (PKS) sequences. Thus, *K*. *mikimotoi* was thought to have several genes encoding PKS metabolites and to likely produce toxin-like polyketide molecules. Of all the sequences, approximately 30 encoded eight PKS genes, which were remarkably similar to those of *Karenia brevis*. Our phylogenetic analyses showed that these genes belonged to a new group of PKS type-I genes. Phylogenetic and active domain analyses showed that the amino acid sequence of four among eight *Karenia* PKS genes was not similar to any of the reported PKS genes. These PKS genes might possibly be associated with the synthesis of polyketide toxins produced by *Karenia* species. Further, a homology search revealed 10 contigs that were similar to a toxin gene responsible for the synthesis of saxitoxin (*sxtA*) in the toxic dinoflagellate *Alexandrium fundyense*. These contigs encoded A1–A3 domains of *sxtA* genes. Thus, this study identified some transcripts in *K*. *mikimotoi* that might be associated with several putative toxin-related genes. The findings of this study might help understand the mechanism of toxicity of *K*. *mikimotoi* and other dinoflagellates.

## Introduction

*Karenia mikimotoi* [(Miyake et Kominami ex Oda) G. Hansen et Moestrup (formerly *Gyrodinium aureolum*, *G*. cf. *aureolum*, G. type-’65, *G*. *nagasakiense*, and *G*. *mikimotoi*) is a harmful dinoflagellate and forms massive blooms in coastal waters of the temperate regions in Europe [1–4] and East Asia [5,6], especially west Japan [7]. Blooms of this species occasionally cause massive mortality of fish and invertebrates, thereby resulting in remarkable damage to fisheries; for example, in 1998, economic losses estimated at US$ 40 million were reported in Hong Kong [8] and, in 2012, economic losses of at least US$ 15 million were reported in Japan [9].

*K*. *mikimotoi* compounds that have toxic effects on marine organisms have been previously reported. Some researchers reported that this species likely inhibits the growth of other algae by producing lipids, sterols, and/or polyunsaturated fatty acids [10,11]. Zou et al. (2010) reported zooplankton mortality due to a hemolytic compound produced by *K*. *mikimotoi* and indicated that the mortality was caused via direct contact between the two organisms [12]. Yasumoto et al. (1990) isolated two toxins—a glycolipid (1-acyl-3-digalactosylglyercol) and a fatty acid (octadecapentaenoic acid)—from *K*. *mikimotoi* and suggested that these compounds had adverse effects on fish [13]. Satake et al. (2002, 2005) isolated hemolytic and cytotoxic compounds, namely, gymnocin A and B, from *K*. *mikimotoi*; the chemical structures of these compounds are partly similar to those of a neurotoxin, namely, brevetoxin (BTX), produced by *Karenia brevis* [14,15]. The mechanisms associated with fish and invertebrate mortality caused by *K*. *mikimotoi* have been extensively studied; however, the significant causative agents responsible for the massive mortality of marine organisms have not yet been identified.

Recent advances in sequencing technology have enabled the generation of genome-wide data from non-model organisms; this method has been used for exploring toxin-related genes in marine organisms. Over the last several years, RNA sequence data of *K*. *brevis*, as well as those of *Alexandrium fundyense*, *Alexandrium ostenfeldii*, *Heterocapsa circularisquama*, *Gambierdiscus polynesiensis Gambierdiscus australes*, *Gambierdiscus belizeanus*, and *Azadinium spinosum*, have gradually been accumulated [16–25]. Numerous transcripts were obtained from these dinoflagellates, and several sequences were thought to encode various polyketide molecules [26]. Algal toxins such as BTXs consist of polyketide molecules that are putatively produced by polyketide synthases (PKSs) [26]. In all, 21 fragments (partial sequences) of PKS genes were isolated from a BTX-producing marine dinoflagellate *K*. *brevis* by using polymerase chain reaction (PCR) and known ketoacyl synthase domain-specific primers [27–29]. The complete sequence of PKS gene has also been characterized in other dinoflagellates such as *A*. *ostenfeldii* and *Heterocapsa triquetra* [30].

In addition to polyether toxins, purine alkaloid toxins and saxitoxin (STX) and its analog gonyautoxins (GTXs) produced by marine dinoflagellates and several freshwater cyanobacteria have been studied [31]. The STX biosynthetic pathway in freshwater cyanobacteria has been well investigated; the genes involved in this pathway were identified as *sxt* [32]. Furthermore, recent studies have shown that marine dinoflagellate species such as *Alexandrium fundyense*, *A*. *minutum*, *A*. *tamarense*, *Pyrodinium bahamense*, and *Gymnodinium catenatum* contain the cyanobacterial *sxt* gene orthologs in their genomes [33,34].

Although there is sufficient information on toxin-related genes of microalgae, sequence data of *K*. *mikimotoi*, an important harmful species in fisheries, is limited to comprehensively understand its toxicity. This study aimed to construct an RNA-sequence library for *K*. *mikimotoi* and to identify toxin-related genes. We identified few transcripts of toxin-related genes. Our results might form the basis for further studies on dinoflagellate toxicity.

## Materials and Methods

### Cell collection and mRNA extraction

We used a clonal axenic strain of *K*. *mikimotoi* (strain Km69-9) isolated from Saeki Bay, Japan (32.98N, 131.98E) in May 2012. No specific permits were required for the described field studies, since the location is not privately owned or protected in any way, and the field studies did not involve endangered or protected species. Axenic strain of *K*. *mikimotoi* was established using the following methods. The *K*. *mikimotoi* cells were cultured in F/2 medium [35] enriched with 10 nM Na_2_SeO_3_ and 4.13 mM Tris (final concentration; 0.5 g/L, pH = 7.5) but without silicate and CuSO_4_. A 0.05 mL antibiotic mixture AM9 (which contains 250 μg dihydrostreptomycin; 250 u potassium penicillin G; 7.5 u polymyxin B sulfate; 12.5 u tetracycline; 12.5 u neomycin; and 2.5 μg chloramphenicol) [36] was added to 1 mL of F/2 medium, and the cells were cultured in the antibiotics medium for 5 days under the conditions described above. After the treatment, living cells were transferred to F/2 medium and cultured. The cells in that culture were then treated with two cycles of the swimming method [37] to produce an axenic condition, i.e., free from bacteria. Subsequently, 0.5 mL of the culture was grown in 10 mL of ST10^-1^ medium (0.5% [wt/vol] tryptone and 0.05% [wt/vol] yeast extract) under the conditions described above to assess the existence of bacteria [37, 38]. Cultures confirmed as axenic were checked purity by direct observations. The candidate axenic cultures (1 mL) were stained with SYBR Gold (1.0 × 10^−4^ dilution of the commercial stock; Life Technologies, Carlsbad, CA) for 15 min in the dark at room temperature and filtered through 0.2 μm Black Nuclepore filters (GE Healthcare Japan, Tokyo, Japan). Subsequently, the filters were mounted on glass slides with a drop of low-fluorescence immersion oil and then covered with another drop of oil and a cover slip. The slides were viewed at a magnification of 1,000× under an epifluorescence microscope (by using a WBV fluorescence cube: excitation, 400 to 440 nm; emission, 475 nm; dichroic mirror, 455 nm; BX50, Olympus, Tokyo, Japan). The presence of bacterial cells was assessed by observing all fields of view. Cultures subjected to the above treatments with no bacteria were defined as axenic *K*. *mikimotoi* culture in this study. The treated cells were then cultured in SWM3 medium [39, 40] and used for further experiments.

Cultures were maintained in 100-mL Erlenmeyer flasks containing 50 mL modified SWM-3 medium [41] having salinity of 32 at 25°C under 300 μmol photons·m^-2^·s^-1^ of white fluorescent irradiation and a 12-h:12-h light:dark cycle [light period, 0600–1800 h local time].

Various mRNA sequences were collected by preparing two different cultures for *K*. *mikimotoi* strain Km69-9—one was the logarithmic growth phase culture (A) and the other was late-stationary culture exposed to heat shock stress at 37°C for 3 min (B). Each 50 mL of the exponential phase or late-stationary phase cultures was 10-fold concentrated using a 3-μm nominal pore size polycarbonate membrane filter (Nuclepore, Whatman, Kent, UK). Next, 5 mL RNase inhibitor (475 mL EtOH + 26.75 g phenol) was added to the cell suspension and vigorously mixed. The cells were pelleted after centrifugation at 2,500 *g* for 5 min at room temperature. The cell pellets were stored at -80°C until RNA extraction. Total RNA was extracted from the cell pellets for both the cultures by using the RNeasy Plant Mini Kit combined with DNase (Qiagen, Valencia, CA). The qualities and quantities of the total RNA samples were determined using an automated electrophoresis system, Experion^TM^ (Bio-Rad Laboratories, Hercules, CA).

### RNA sequencing

Before the sequencing library was prepared, rRNA was depleted from 2.5 μg of total RNA samples by using Ribo-Zero™ rRNA Removal Kit (Plant Seed/Root; Epicentre, Madison, WI, USA). rRNA removal was confirmed using Agilent RNA 6000 Nano Kit (Agilent Technologies, Santa Clara, CA) by using a 2100 Bioanalyzer (Agilent Technologies, Santa Clara, CA). For RNA sequencing, double-stranded cDNA libraries were constructed using TruSeq Stranded mRNA Sample Preparation Kit (Illumina, San Diego, CA), and two samples for each growth phase were independently indexed. The condition of the obtained libraries was validated using an Agilent DNA 1000 Kit (Agilent Technologies, Santa Clara, CA) by using a 2100 Bioanalyzer, and their concentrations were measured using Qubit® 2.0 Fluorometer (Life Technologies, Carlsbad, CA). The validated library for each sample was mixed and denatured with NaOH, and then diluted to 20 pM by using the hybridization buffer included in the MiSeq Reagent Kit v2 (Illumina). The final library was subsequently loaded to a 500-cycle MiSeq reagent cartridge for sequencing by using MiSeq (Illumina) platform having sequenced runs of 2 × 250 paired-end reads.

### Data assembly and annotation

Raw sequencing data processed using the Illumina MiSeq platform were deposited in DDBJ [accession numbers: DRA002948 (sample A) and DRA002947 (sample B)]. The reads were assembled into transcript contigs by using the all-in-one package, Rnnotator [42], with nonP mode and the other default settings, which performs read pre-processing, *de novo* assembly, and contig post-processing. We constructed three types of assemblies for samples A, B, and their combined database to maximize gene discovery. All the transcript contigs were used for annotation against the NCBI non-redundant (NR) protein database [43] and Kyoto Encyclopedia of Genes and Genomes database [44] by using BLASTX search [45] with the cutoff E-value of 1e^-5^. Annotated species names were obtained based on the best hits of NR. The KEGG functional distributions were calculated based on the KEGG Orthology (KO) identifiers obtained from the results of BLASTX to KEGG. In addition, the alignment regions of the best hit DNA sequences of BLASTX to NR were translated into the amino acid sequences. Transcriptome completeness was confirmed using the Core Eukaryotic Genes Mapping Approach (CEGMA) tool by using a subset of 248 core eukaryotic genes (CEGs) under the default settings of the software against the *K*. *mikimotoi* amino acid sequence database [46]. The Pfam [47] domain search against the *K*. *mikimotoi* amino acid sequence database was performed using HMMER3 [48] with cut_ga and domtblout options. The sequences, including PKS gene-related Pfam domains (PF00109: ketoacyl synthase (KS), PF08659: ketoreductase [KR], PF00698: acyltransferase [AT], and PF01648: acyl carrier protein [ACP]) were extracted from the PFAM database (http://pfam.xfam.org). Further, the KS-related PKS genes were identified using HMMER analysis with the HMM profile constructed from aligned amino acid sequences of 20 dinoflagellate KS domain.

### Sequencing of incomplete PKS genes

Total RNA was extracted from the cell pellet of *K*. *mikimotoi* strain Km69-9 by using an RNeasy Mini Kit (Qiagen, Valencia, CA). cDNA was synthesized using SuperScript III Reverse Transcriptase kit (Life Technologies, Carlsbad, CA) by using the oligo-dT primer. Subsequently, 0.5 μL cDNA samples were added to 19.5 μL PCR mixture that included *K*. *mikimotoi* PKS gene-specific primers (Table 1). PCR was performed under the following conditions: 1 cycle at 94°C for 2 min; 25 cycles each consisting of 94°C for 30 s, 55°C for 30 s, and 72°C for 1 min; and a final step at 72°C for 10 min. Amplicons were purified using a QIA quick PCR Purification Kit (Qiagen, Valencia, CA) and ligated into the pGEM-T Easy Vector (Promega, Madison, WI). Sequencing reactions were performed using universal primers (U19 or M13 reverse) and BigDye Terminator v3.1 (Life Technologies, Carlsbad, CA) and an ABI PRISM 3130xl DNA Analyzer (Life Technologies, Carlsbad, CA). The generated sequences were manually assembled into contigs.

**Table 1:** Primers used in the RT-PCR analysis.

Name	Sequence 5′ -3′	Orientation	Target transcripts
P1008-2	AGGATAGCATCTTAAGGATGGG	Forward	Km_PKS_5
P1008-3	TAGATTGCCAGAACCATGGC	Reverse	Km_PKS_5
P1008-4	TTGGATGACACAGGCTATGC	Forward	Km_PKS_5
P1008-5	CGTCAATGAGCCACGAAGAG	Reverse	Km_PKS_5
P2006-2	CACAGTGCTTCAATTTGTGG	Forward	Km_PKS_6
P2006-3	TCGATTTGCATGCTTGCTCC	Reverse	Km_PKS_6
P2006-4	TTCGAGTGTCATGGAACTGG	Forward	Km_PKS_6
P2006-5	TTCCTCAAATGAAGGTTCGGG	Reverse	Km_PKS_6
P4825-1	ACAAGTGCATGCTGATGTCC	Forward	Km_PKS_7
P4825-2	GCCATGTAAGAGGCCAAATG	Reverse	Km_PKS_7
P4825-3	CCAAGCCGTCATTAAGGAGG	Forward	Km_PKS_7
P4825-4	TCTGCACAGTCAATGTCACC	Reverse	Km_PKS_7
P6842-1	CCCTGAGGGAGGCAAATATC	Forward	Km_PKS_8
P6842-2	TACCTGTGCTCATAAGCAGG	Reverse	Km_PKS_8
P6842-3	ATGGTAGGAAGAGGGTCTGG	Forward	Km_PKS_8
P6842-4	CCTGATGAAAGCCATGAACC	Reverse	Km_PKS_8

### Phylogenetic analysis and comparison of active motif of PKS_KS domain

Amino acid sequences of PKS gene and fatty acid synthase (FAS) gene, which were involved in PKS, encoding the PKS_KS domain were obtained from GenBank. Six bacterial type-I PKSs, 5 animal FASs, 4 chlorophyte PKSs, 5 haptophyte PKSs, 5 apicomplexa PKSs, 19 dinoflagellate PKSs, 7 identified *K*. *mikimotoi* PKSs, and 4 type-II PKS genes were used in the phylogenetic analysis. Amino acid sequences were automatically aligned using ClustalW [49], and the resulting alignment was manually refined. Next, only the KS domains in each sequence were picked from the entire PKS/FASs sequences.

Phylogenetic trees were constructed according to the KS domain by using the maximum likelihood (ML) method by using MEGA 6.0 software [50]. The LG model (with gamma distributed rate across sites and amino acid frequencies estimated from the actual sequence data), were selected using the build-in best protein model selection and was used for the construction of the ML tree.

Three sequences of KS active domains in above PKSs were aligned. Conserved amino acids were picked from each domain by using sequence logo tool (Weblogo: http://weblogo.berkeley.edu/logo.cgi).

## Results

### Sequence assembly and contig number and length

Two cDNA samples prepared from *K*. *mikimotoi* samples A and B were sequenced using the Illumina sequencing platform. After the sequences were pre-processed and filtered using the Illumina platform, approximately 10 million reads were obtained for each sample. Rnnotator [41] was used to assemble the paired-end reads into transcript contigs and to measure the expression level. Finally, 153,406 transcript contigs with average lengths of 687 nt were generated as combined database from samples A and B (Table 2).

**Table 2:** Statistical analysis of assembled contigs.

Sample	A	B	Combined
Min length (nt)	181	177	181
Max length (nt)	5,338	5,638	7053
Average length (nt)	566	562	687
N50 (nt)	646	648	900
Total length (nt)	66,963,871	72,455,377	105,347,406
Total contig number	118,405	128,875	153,406

BLASTX top hit to KEGG was used, and 39,660 (33.4%), 41,360 (32.1%), and 51,807 (33.8%) contigs for samples A, B, and the combined database, respectively, were annotated. Functional distributions of these data were similar to each other (Table 3).

**Table 3:** The KEGG functional distributions of contigs in each database.

	Database		
Function	A	B	Combined
Amino acid metabolism	5.6%	5.4%	5.5%
Biosynthesis of other secondary metabolites	1.7%	1.7%	1.7%
Carbohydrate metabolism	7.8%	7.8%	7.2%
Cardiovascular diseases	1.9%	1.9%	2.1%
Cell communication	4.3%	4.4%	4.6%
Cell growth and death	6.3%	6.6%	6.5%
Cell motility	2.6%	2.6%	2.8%
Energy metabolism	5.8%	5.6%	5.0%
Environmental adaptation	5.1%	5.3%	5.4%
Folding, sorting and degradation	6.7%	6.8%	6.9%
Glycan biosynthesis and metabolism	1.8%	1.8%	1.9%
Lipid metabolism	4.8%	4.8%	4.9%
Membrane transport	1.1%	1.1%	1.2%
Metabolism of cofactors and vitamins	3.2%	3.2%	3.1%
Metabolism of other amino acids	2.5%	2.3%	2.2%
Metabolism of terpenoids and polyketides	2.1%	2.0%	1.9%
Nucleotide metabolism	3.1%	3.1%	2.9%
Replication and repair	2.0%	1.9%	2.0%
Signal transduction	10.8%	11.0%	11.4%
Signaling molecules and interaction	0.6%	0.7%	0.7%
Transcription	3.3%	3.2%	3.1%
Translation	6.2%	6.1%	6.1%
Transport and catabolism	7.5%	7.5%	7.5%
Xenobiotics biodegradation and metabolism	3.3%	3.3%	3.4%
Total contig number	39660	41366	51807

**Fig 1:**
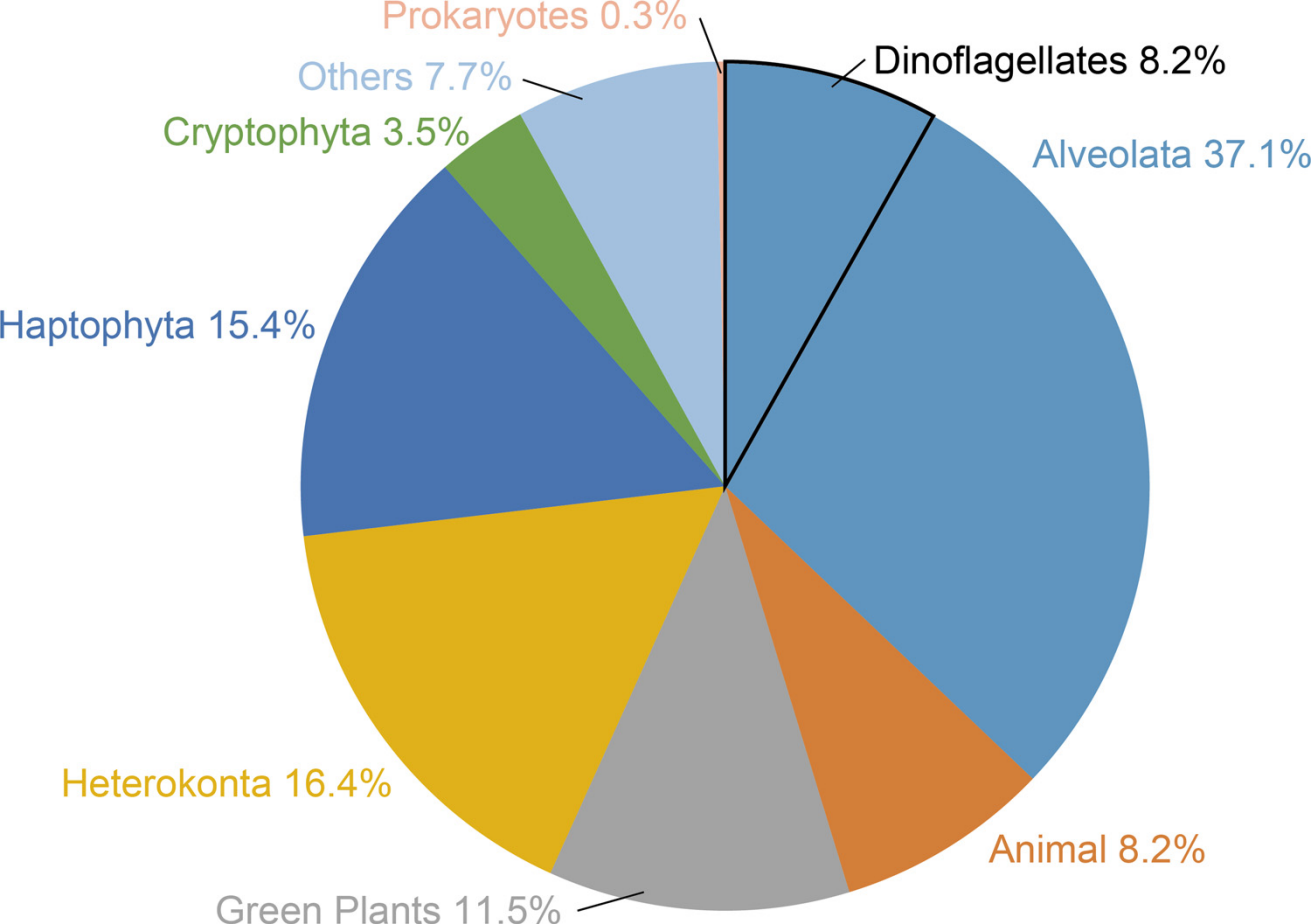
Taxonomic distribution graph of BLASTX top hit against KEGG in *K*. *mikimotoi* contigs. BLAST top hit organisms were grouped into eight taxa as follows: Alveolata, Animals, Green Plants, Heterokonta, Haptophyta, Cryptophyta, Prokaryotes, and Others. Dinoflagellates were enclosed by black line within the group Alveolata.

Analysis of BLASTX top hit to *K*. *mikimotoi* from the combined database showed that 37.1% of them matched with alveolata organisms, which include dinoflagellates (8.2%; Fig 1). Most of these contigs had homology to eukaryote genes except genes of uncultured bacterium (0.3%; S1 Table). Completeness of the *K*. *mikimotoi* transcriptome dataset was determined using the CEGMA tool [46]. Thus, 190 (77%) among the 248 CEGs were found.

### Identified PKS sequences in *K*. *mikimotoi*

A keyword search of “polyketide synthase” to the BLASTX top hit database of the combined contigs revealed 208 putative PKS-encoding contigs (Table 4). The Pfam domain search on HMMER3 against combined amino acid dataset showed that 86, 31, 118, and 8 contigs matched with KS, KR, AT, and ACP, respectively (Table 4). Further, conserved sequences of KS domains in dinoflagellates matched 114 contigs according to HMMER analysis (Table 4).

**Table 4:** Detected contig numbers in PKS genes or PKS domains search.

Gene/domain	PfamID	Method	Contig numbers
Polyketide synthase	-	Keyword search*	208
Ketoacyl synthase (KS)	PF00109	Domain search**	86
Ketoreductase (KR)	PF00698	Domain search**	31
Acyltransferase (AT)	PF08659	Domain search**	118
Acyl carrier protein (ACP)	PF01648	Domain search**	8
Dinoflagellate ketoacyl synthase	-	Domain search**	114

* Keyword search was conducted against BLAST top hit tables of all contig databases of *K*. *mikimotoi*

** Domain search was performed against amino acid sequence databases of *K*. *mikimotoi* by using HMMER3.

Contig sequences of 86 KS domains identified by Pfam domain search have not yet been recorded in the Gene Bank. Moreover, completed CDSs were not detected in any of these contigs. Local protein BLAST search against the amino acid database of the combined contigs in *K*. *mikimotoi* revealed that the sequences of approximately 30 contigs were similar to those of previously identified eight full-length PKS genes in *K*. *brevis* [29] (Table 5). Manual assembling of these contigs revealed 4 PKS genes with complete open reading flames (Km_PKS_1 to 4) and 4 partial PKS genes (Km_PKS_5 to 8) in *K*. *mikimotoi*. The former four genes encoded single catalytic domains for each gene; Km_PKS_1 gene only encoded the KR domain and the others, Km_PKS_2 to Km_PKS_4, encoded the KS domain. A long sequence fragment of the remaining four genes (Km_PKS_5 to Km_PKS_8) was detected by conducting reverse transcriptase-PCR and subsequent sequencing by using primers that were designed based on the corresponding contig sequences. Thus, over 2,400 bases of each gene were detected, and all of them encoded the KS domain. Finally, the 8 assembled PKS transcripts of *K*. *mikimotoi* were reanalyzed using BLAST search; they had significant homology to *K*. *brevis* PKS gene with high level of sequence identities (>74%; Table 5).

**Table 5:** Summary of PKS genes in *Karenia mikimotoi*.

	*K*. *mikimotoi* PKS Accession No	Contig length	Amino acids	Compleate CDS	Predicted function	*K*. *brevis* PKS Accession No	e-value	Identity
Km_PKS_1	LC022746	1815	516	Yes	KR	EF410009	0.0	82%
Km_PKS_2	LC022747	3139	941	Yes	KS	EF410010	0.0	82%
Km_PKS_3	LC022748	3145	945	Yes	KS	EF410011	0.0	84%
Km_PKS_4	LC022749	3044	881	Yes	KS	EF410013	0.0	74%
Km_PKS_5	LC022750	3101*	1034*	No	KS	EF410006	0.0	81%
Km_PKS_6	LC022751	2537*	792*	No	KS	EF410007	0.0	86%
Km_PKS_7	LC022752	2817*	938*	No	KS	EF410008	0.0	75%
Km_PKS_8	LC022753	2455*	816*	No	KS	EF410012	0.0	74%

* shows partial sequence lengths

### Phylogenetic analysis and comparison of the active motifs of PKS_KS genes

**Fig 2:**
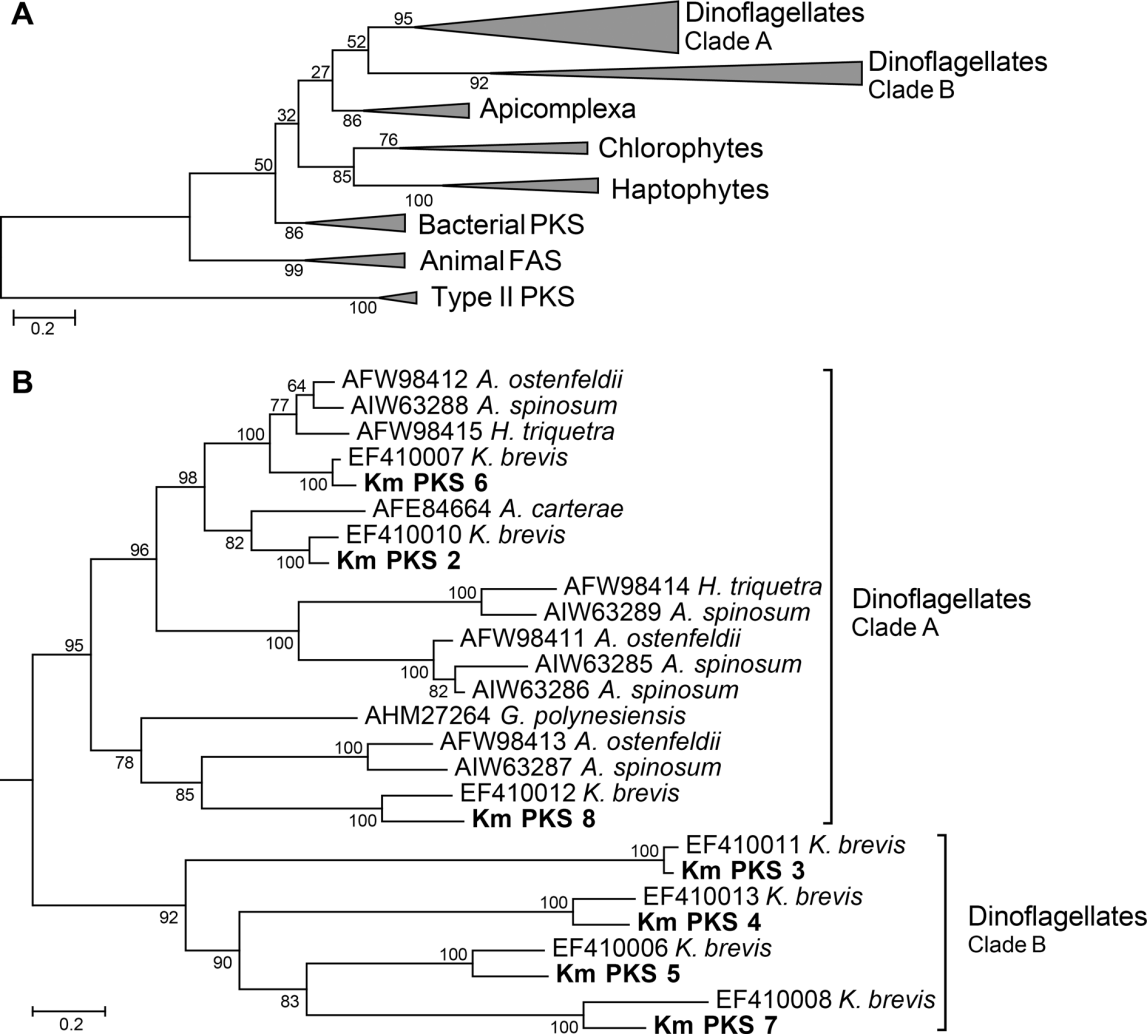
Phylogenetic analysis of the KS domains in type-I and type-II PKS and fatty acid synthase (FAS). (A) KS domains of 59 taxa were analyzed using the maximum likelihood (ML) method. (B) Focused ML-tree of PKS_KS domains of dinoflagellate. Bootstrap values (%) from 100 samples are shown at the nodes in each tree. ML distance scale bars are shown under the trees. Bars with descriptions on the right show clades of each type of PKS molecules.

**Fig 3:**
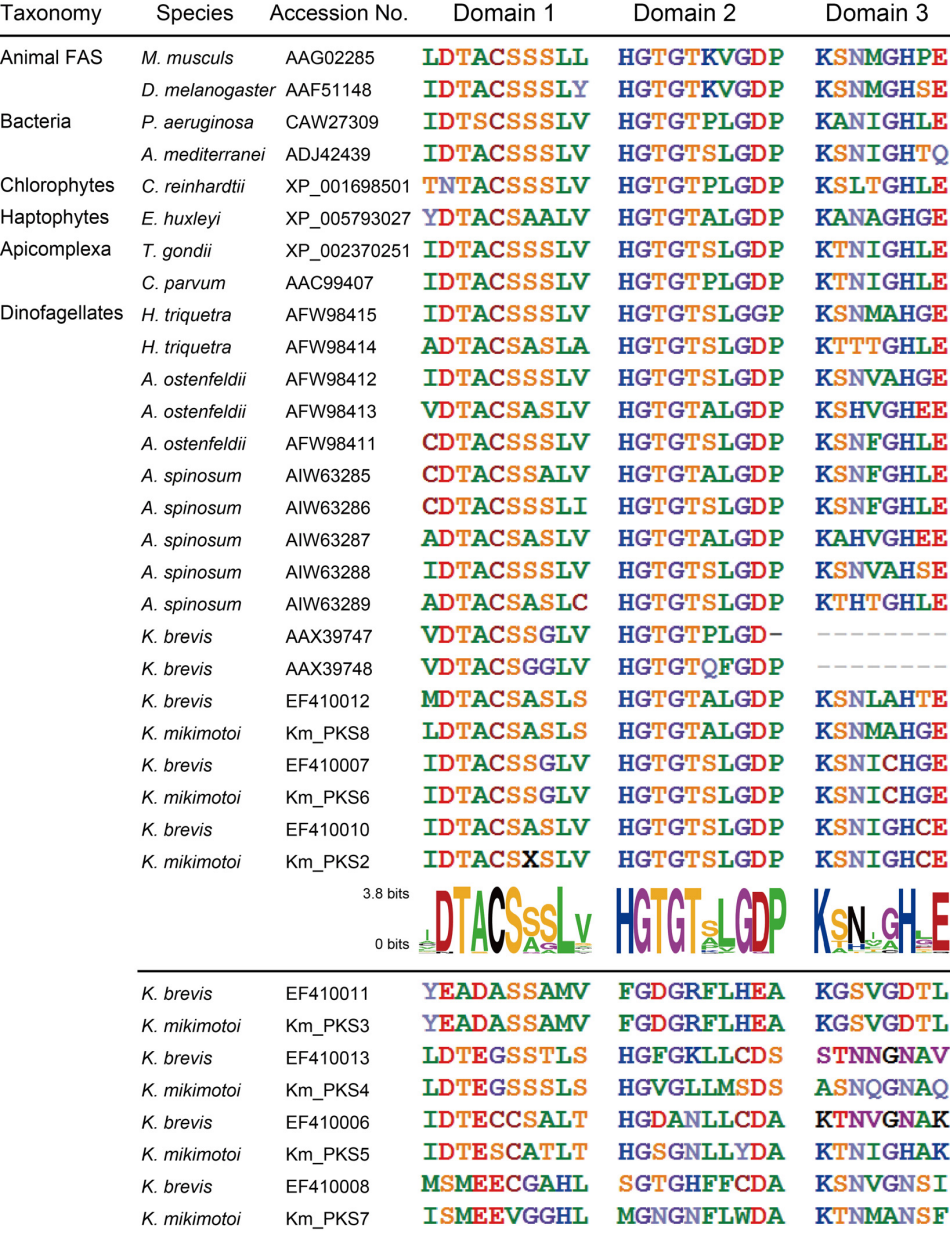
Comparison of amino acid sequences of KS active site in various PKSs. This analysis focused on three KS domains of dinoflagellate, Apicomplexa, Haptophytes, and others. The sequence logos provided the conservation of each amino acid sequence against PKSs mentioned above the middle line.

Phylogenetic analysis for the KS domain of PKS/FASs in *K*. *mikimotoi* and other organisms showed that they were separated into seven clades (Fig 2A). The animal FASs and type-II PKSs formed independent clades with high bootstrap value (99% and 100%). Haptophytes, chrolophytes, bacterial, apicomplexa, and dinoflagellate type-I PKSs were separated with bootstrap values of 100%, 76%, 86%, 86%, and 52%, respectively. In the dinoflagellate PKS clade, each of the seven different PKSs of *K*. *mikimotoi*, Km_PKS_2 to Km_PKS_8, formed individual monophyletic clades to *K*. *brevis* PKSs with high bootstrap values (100%; Fig 2B). Dinoflagellate PKSs were separated into two clades (Fig 2B, clades A and B) and, interestingly, eight *Karenia* PKSs (Km_PKS_3, Km_PKS_4, Km_PKS_5, Km_PKS_7, EF410006, EF410008, EF410011, and EF410013) in clade B were independent from other dinoflagellate PKSs with high bootstrap values (92%). KS active domain comparison analysis within type-I PKS showed that most of their first and second active domains have DTACS-motif and HGTGT-motif, respectively (Fig 3); however, the eight *Karenia* PKSs (Km_PKS_3, Km_PKS_4, Km_PKS_5, Km_PKS_7, EF410006, EF410008, EF410011, and EF410013) mentioned above did not. Although the eight PKSs are type-I PKS_KS, they have different amino acid sequences in their active domains (Fig 3).

### Identified saxitoxin biosynthetic (*sxt*) genes in *K*. *mikimotoi*

The protein BLAST top hit analysis showed that 10 out of all the contigs were similar to *sxtA* sequences of *A*. *fundyense* (Table 6, S1 Text). Six contigs among them were similar to signal peptides, A1–A3 domains and C-terminal fragment of shorter *sxtA* genes. The other four contigs had homology with the signal peptide and A1–A3 domains of longer *sxtA* genes. Each contig was similar to *sxtA* genes in *A*. *fundyense* with e-values ranging from 2E^-2^ to 2E^-39^ and had identity from 39% to 65% (Table 6).

**Table 6:** Summary of *sxtA* genes in *Karenia mikimotoi*.

*Sequence* names†	Contig length	Amino acids	Homology genes	Homology domain	*Alexandrium fundyense sxtA* genes Accession No	e-value	Identity
Km_sxtA_1	334*	102*		Signal peptide, A1	JF343238	4E-21	50%
Km_sxtA_2	314*	104*	*Alexandrium*	A1		2E-27	49%
Km_sxtA_3	304*	101*	*fundyense*	A1, A2		1E-25	50%
Km_sxtA_4	313*	104*	shorter sxtA	A2		5E-26	51%
Km_sxtA_5	303*	101*	gene	A2, A3		4E-14	39%
Km_sxtA_6	446*	130*		C-terminal		2E-08	41%
Km_sxtA_7	315*	30*	*Alexandrium*	Signal peptide, A1	JF343239	0.022	65%
Km_sxtA_8	447*	149*	fundyense	A1, A2		2E-39	48%
Km_sxtA_9	342*	114*	longer sxtA	A2		2E-24	50%
Km_sxtA_10	463*	154*	gene	A2, A3		2E-17	52%

† All sequences are provided in S1 Text

* shows partial sequence lengths

## Discussion

The total read length of the transcripts isolated in the present study was calculated to be approximately 105 Mb in combined dataset from samples A and B. The nuclear genome size of the dinoflagellate species was estimated to be 1.5–185 Gb [51]. Of the 18–23 Gb genome size of *Heterocapsa triquetra*, 89.5% was estimated to be noncoding DNA [52]. Thus, the total mRNA sequence length of dinoflagellate species ranges from several hundred mega base pairs to a few dozen giga base pairs; however, the mRNA sequence length of the dinoflagellate species used in this study was smaller than the reported mRNA lengths. Further, N50 statistics was about 900 nt. This value is considerably small, indicating that sufficient mRNA sequences were not assembled by RNA sequencing.

By KEGG analysis, functions of over 33.8% contigs were annotated. These transcripts were annotated to diverse functional genes (Table 3). Functions of signal transduction, carbohydrate metabolism and transport, and catabolism were more frequent than other functions. However, there seemed to be no difference among the three database sets. The quality of the present samples might be similar in terms of diversities for transcripts.

Taxonomic distribution of BLASTX top hit analysis to *K*. *mikimotoi* contigs showed that ca. 37% of the matched taxon were alveolata that includes dinoflagellates (8.2%). Other contigs were matched with diverse taxa of eukaryotes. In general, dinoflagellates have huge size genome, and databases for dinoflagellates are still incomplete. Therefore, the results obtained in the present study might reflect the limited available databases. Nonetheless, for future studies, more huge genome and transcript databases of various dinoflagellates need to be constructed.

The CEGMA tool analysis showed that 77% of the CEGs were present in the *K*. *mikimotoi* transcriptome dataset. Similar values were reported by previous dinoflagellate transcriptome studies as follows: *Karlodinium micrum*, 74%; *K*. *brevis* SP1, 84%; SP2, 82%; and Wilson, 81%; *Gambierdiscus australes*, 84%; *Gambierdiscus belizeanus*, 73%; and *A*. *spinosum*, 94% (23, 24, 25). Thus, the quality of the *K*. *mikimotoi* transcriptome data in this study seemed to be comparable to those of previous reports.

In this study, we identified eight different PKS genes of *K*. *mikimotoi* that were remarkably similar to those [29] of *K*. *brevis*, which is a relative species of *K*. *mikimotoi* by contig assembly and PCR-sequencing methods. Further, phylogenetic analysis indicated that each PKS had an ortholog in each *Karenia* species. A previous study showed that *K*. *mikimotoi* has only four orthologs of the characterized PKSs of *K*. *brevis* [29]. However, we showed that *K*. *mikimotoi* harbors four additional orthologs of *K*. *brevis* PKSs. Any ortholog of the remaining 21 partial PKS transcripts from *K*. *brevis* culture [27,28] were not detected in *K*. *mikimotoi* transcripts, even though more than 200 PKS-related contigs were detected in this study (Table 4). The *K*. *mikimotoi* culture used in the present RNA sequence analysis was considered to be in axenic condition (see materials and methods), and BLASTX top hit to *K*. *mikimotoi* contigs against KEGG showed that very few prokaryote genes were included in this dataset (Fig 1). However, *K*. *brevis* cultures used for sequence analysis in previous studies harbored associated bacteria (Table 7) [27–29]. Therefore, some of the 21 PKS sequences of *K*. *brevis* might be of bacterial origin or bacterial chimera sequences. Nonetheless, numerous PKS-related contigs are uncharacterized in *K*. *mikimotoi*, and other novel PKS genes would be present.

**Table 7:** Reports of PKS transcripts in genus *Karenia*.

	This study	Snyder et al. 2005	Monroe et al. 2008	López-Legentil et al. 2010
Method	RNA sequencing	EST serch	PCR	PCR
Origin	*K*. *mikimotoi*	*K*. *brevis*	*K*. *brevis*	*K*. *brevis*
Associated bacteria	not include	include	include	include
No. PKS genes	8	3	8	18
Poly A	detected	nd	nd	detected
Single PKS catalytic domain	detected	nd	nd	detected

nd: no data

Previously known PKSs have been categorized into three groups: types I, II, and III [53]. PKSs include several domains such as ACP, AT, KR, and KS to allow the catalysis of polyketide synthesis. Typical type-I PKSs consist of multi-domain structures, whereas type-II PKSs have a single catalytic domain [53]. However, the type-I PKSs of *K*. *brevis* encode a single catalytic domain [29], consistent with the findings of this study. On the basis of BLAST search and phylogenetic analysis (Fig 1), the complete coding sequences of the four PKS transcripts of *K*. *mikimotoi*, Km_PKS_1 to Km_PKS_4, were grouped as type-I PKS, in which only a single catalytic domain was found. A recent study found similar results that PKS transcripts of other dinoflagellates, i.e., *A*. *ostenfeldii*, *H*. *triquetra*, and *Azadinium spinosum*, were classified as type-I PKS, but they only had a monofunctional domain [24, 54]. Thus, dinoflagellate type-I PKS that have unique features might be stratified into a different group in type-I PKS.

Polyketides include a diverse class of secondary metabolites that are produced by many organisms [55]. The dinoflagellate *K*. *brevis* produces cyclic polyether compounds—BTXs, a kind of polyketide molecules—that were found to be neurotoxic to shellfish around the coastal area bordering the Gulf of Mexico [56]. The biosynthetic pathway of BTXs has been reported; however, their genetic pathways have not yet been completely elucidated. BTXs are polyether ladders that are uniquely found in dinoflagellates [26]. *K*. *mikimotoi* also produce polyether ladder polyketide, i.e., gymnocin A and B, whose structures are similar to those of BTXs [14,15]. In this study, seven PKS orthologs identified in *K*. *mikimotoi* had KS domain; hence, we constructed ML tree of the KS domain of PKS genes (PKS_KS) by using phylogenetic analysis. The four *Karenia* PKS orthologs (including Km_PKS_3, Km_PKS_4, Km_PKS_5, and Km_PKS_7) are grouped into different clades (clade B) of other dinoflagellate PKSs (clade A; Fig 2B). The four orthologs also have different sequences from those of other PKSs in the active motif of the KS domain (Fig 3). In a recent study of the toxic dinoflagellate *G*. *polynesiensis*, which biosynthesizes ciguatoxins, was found to have similar contigs as those of the four *Karenia* PKSs by KS domain search analysis [22]. Additionally, parts of PKS genes were only present in *Karenia* and *Gambierdiscus* species [25]. Interestingly, the structures of ciguatoxins also consist of a polyether ladder [25, 26]. The biosynthetic mechanisms of polyether ladder polyketides are suggested to be common in dinoflagellates, because their structures are highly similar to each other [26]. Taken together, these findings suggest that the four PKS orthologs in *Karenia* species might possibly be related to the biosynthesis of unique structures as polyether ladder components in polyketide, although this hypothesis needs to be verified further.

Additionally, the HMMER analysis in this study showed that 86 and 114 contigs were identified as the KS domain with PfamID (PF00109) and the HMMs of dinoflagellate KS domain, respectively (Table 4). Twenty-six contigs among them had full-length and conserved active motif of the KS domain. The result of the neighbor-joining tree of various PKS_KS with 26 newly identified contigs showed that they were included in the dinoflagellate PKS clade or Type-II PKS genes (S1 Fig). In some cases, PKS_KS genes of *K*. *brevis* were not detected even in the paraphyletic groups that include the newly identified contigs. There might be diverse uncharacterized PKS_KS genes in *Karenia* species. Further accumulations of sequence data for related organisms might unlock the relationships of those PKS_KS genes in dinoflagellate PKS clades.

Saxitoxin (STX) and its derivative gonyautoxins (GTXs) are other serious toxins of dinoflagellates. Our manual assembling analysis suggested that the 10 contigs of *K*. *mikimotoi* are orthologs of the two *sxtA* genes of *A*. *fundyense* [33]; six are similar to the shorter *sxtA* gene, and the remaining are similar to the longer one. The longer *sxtA* transcript in *A*. *fundyense* has been reported to encode signal peptides at the N-terminal and the subsequent four domains, A1–A4. The shorter *sxtA* gene harbors the same component as that found in the longer one, except that the latter lacks the A4 domain [33]. Some gonyaulacoid dinoflagellates and single species of gymnodinoid, *Gymnodinium catenatum*, are known to produce STX or GTXs, and these dinoflagellates have the longer *sxtA* transcript that includes the A4 domain [33,57]. Therefore, the presence or absence of the A4 domain of the longer *sxtA* transcript is known to determine whether a dinoflagellate produces STX or GTXs. Gymnodinoid dinoflagellates except *G*. *catenatum* are known to lack not only the A4 domain but also the entire longer *sxtA* gene. A horizontal gene transfer might have led to the presence of *sxtA* gene in *G*. *catenatum* [57]. However, in this study, the gymnodinoid dinoflagellate *K*. *mikimotoi* harbored two *sxtA* gene orthologs but not the A4 domain. Therefore, other gymnodinoid dinoflagellates might principally have two *sxtA* genes and lack the A4 domain in the longer *sxtA* transcript. Additionally, another gene, *sxtG*, which also encodes a key enzyme in STX biosynthesis pathway, was not found in *K*. *mikimotoi* transcripts [34]. Orthologs of the A4 domain of *sxtA* and *sxtG* have not been detected in *K*. *mikimotoi* RNA database. Due to the lack of these key enzymes [18,33,34,57], *K*. *mikimotoi* might not be able to produce STX or GTXs. Interestingly, many dinoflagellate species that cannot produce STX were recently shown to lack the *sxtA4* domain [58]. Considering the huge genome sizes of dinoflagellate species, the transcript database constructed in this study is only limited. The present results might reflect the insufficient quantity of the data. Further high-throughput sequence analyses in future studies might reveal the presence or absence of orthologs of the A4 domain in *sxtA* and *sxtG*.

## Conclusions

In this study, we successfully detected partial transcripts of *K*. *mikimotoi*, found many PKS-related contigs, and identified several putative toxin-related genes. Our findings might contribute to the better understanding of toxin biosynthesis in dinoflagellates and the evolution of genes involved in this process. Furthermore, the transcript database generated in this study might be helpful for future dinoflagellate research.

Further studies are warranted to determine the relationships between *K*. *mikimotoi* toxins and their related genes. Although whether gymnocins derived from *K*. *mikimotoi* are toxic to marine water species has not yet been entirely determined, they might still be considered as one of the candidates responsible for fish mortality. If the PKS-encoding genes identified in the present study are found to be associated with gymnocin biosynthesis, they might become an important molecular indicator of bloom toxicity in *K*. *mikimotoi*. Further detailed analysis of the functions of PKSs as well as sequencing analysis for *K*. *mikimotoi* and survey of gymnocin toxicities is necessary.

## Supporting Information

S1 Fig Phylogenetic analysis of the KS domains in type-I and type-II PKS and FAS with identified PKS_KS contigs. PKS_KS domain dataset for ML tree (Fig 1) and 26 newly identified PKS_KS contigs (bold faces) were used in the phylogenetic analysis. Amino acid sequences were aligned using ClustalW and manually refined. Phylogenetic trees were constructed according to the KS domain by using the neighbor joining (NJ) method using MEGA 6.0 software. Bootstrap values (%) from 100 samples are shown at the nodes in each tree. NJ distance scale bars are shown under the trees.
(PDF)

S1 Table Taxonomic distribution of BLASTx top hit to *K*. *mikimotoi* contigs. (XLSX)

S1 Text Contig sequences of shorter and longer *sxtA* in *K*. *mikimotoi*. (DOCX)
