# Supplementary figures and images for: RNA Sequencing Revealed Numerous Polyketide Synthase Genes in the Harmful Dinoflagellate Karenia mikimotoi
Source: PLoS One. 2015 Nov 11;10(11):e0142731. doi: 10.1371/journal.pone.0142731 (PMC4641656; doi:10.1371/journal.pone.0142731)

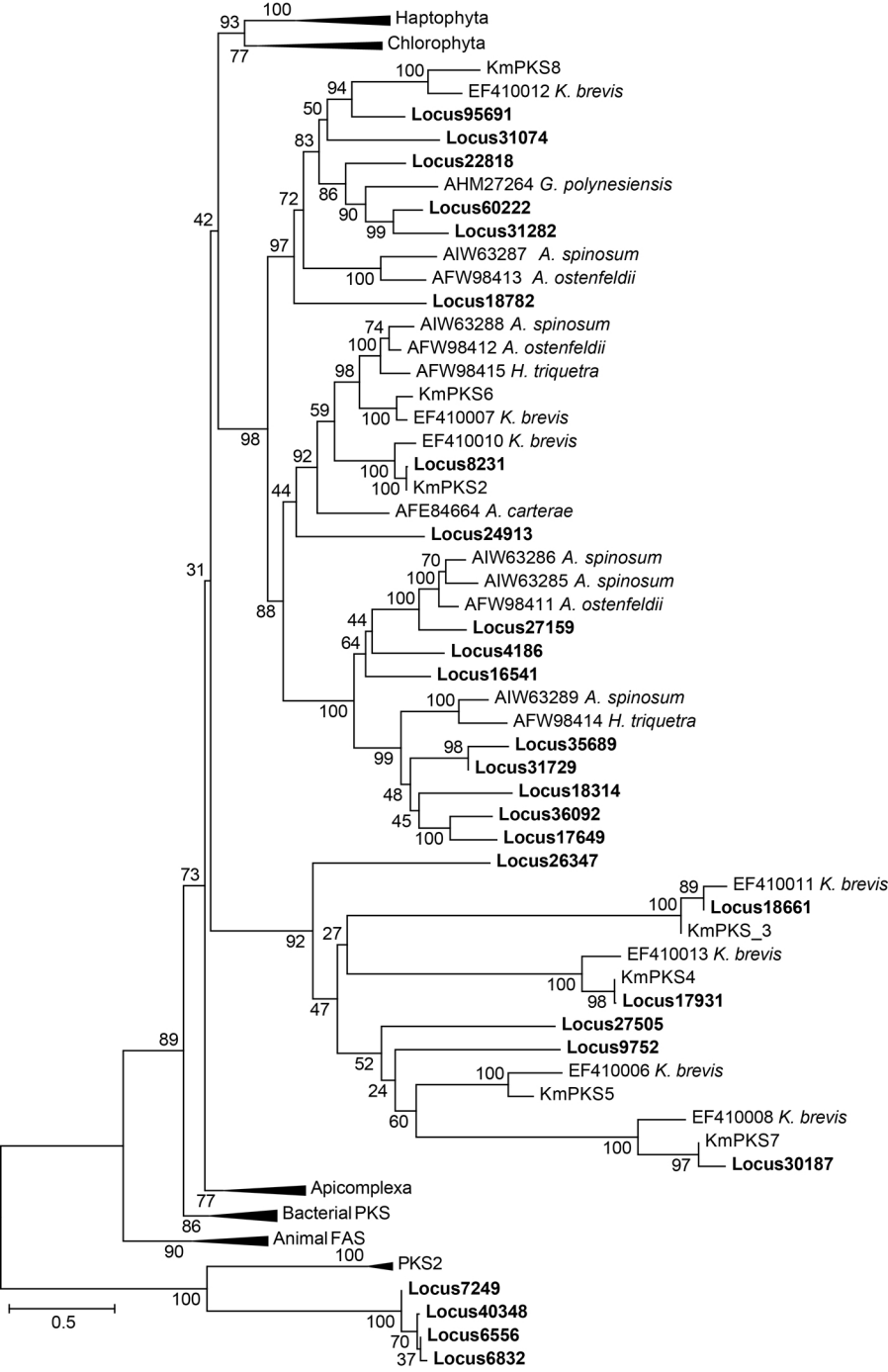

Supplement: S1 Fig — PKS_KS domain dataset for ML tree (Fig 1) and 26 newly identified PKS_KS contigs (bold faces) were used in the phylogenetic analysis. Amino acid sequences were aligned using ClustalW and manually refined. Phylogenetic trees were constructed according to the KS domain by using the neighbor joining (NJ) method using MEGA 6.0 software. Bootstrap values (%) from 100 samples are shown at the nodes in each tree. NJ distance scale bars are shown under the trees. (PDF) [file pone.0142731.s001.pdf]
