# Supplementary material for: RNA Sequencing Revealed Numerous Polyketide Synthase Genes in the Harmful Dinoflagellate Karenia mikimotoi
Source: PLoS One. 2015 Nov 11;10(11):e0142731. doi: 10.1371/journal.pone.0142731 (PMC4641656; doi:10.1371/journal.pone.0142731)
Supplement: S1 Text — (DOCX) [file pone.0142731.s003.docx]

**S1 text. Contig sequences of shorter and longer *sxtA* in *K. mikimotoi*.**

>Km_sxtA_1

GTTTGGCATCTGTGTAAATACTGTCGATTTCCTCGCATAATGGATCTGTTGGATCTAAGTACTTTTCTAATTCATCCAGTTCCGACCCCTCTTTGAGCTTGTATCTGCCTGTGCTTCCATCGAATTCAACGTACTCCAAGATGCTACAAGTTCGCAGAACGATTGCAAGAGGACCTTCTGTTGCACCTGTCTGCCTTGCAAGTTCTGCAGCCGTGGCTCCATCCGGTGCATTTCTCCGCAGCGGCCCAGAAAAATGTGCAACGAATTCCATCAGTAGCGTGCTGCACGTCCAACCATGGGCCAGCATGGGGGCGTTCGTGGCCAGACGAATGCC

>Km_sxtA_2

GTTGGGAACGCCCTTTTGACTCAACTTCAATTTCAGAAGGGTCTTGGCTTCCTCATCATATTCAATGCCAATCATAGTGAGTGGGTATCTGCTCAGTACTTTGCCGCGTGGCGTCTTCTCTTTGATGAACTCATAGGCATGCAACAAGAAATATCCATCGCCACTGCCCGCGTCTGCGAGGTACGACGGTTGCGATGCAAATTCCTCGCCAGCGAAGACTAGGCCAAGGTGATGCATCATATCGCTGAATAAGTTTAAATTTGGAAGACCGCTGTTGACCACGCTTACAAGGTTCTCGGCATGGATCTCCGTCC

>Km_sxtA_3

GTATCCGAAGCGTGGCAATTATTGCCAGACTATGGCACAGTATCTTGTGAAGCGCCCATTCAAAATACGACTCGCTGAGAAATCGGATTTGTCCGAGCTTGTGAGACTAGAAGCTCTGACGTGGGATAAGCACCTGCAAGTACCCGAAAAGGTATTGAGCAGACGCTTGAAAACATCTCCTGCCACTGTATTGGTTTGTGAAGTTGACAAGAAGTTGGTTGGAGTCCTGTACATGCAGAAAATCGAGAGTCTGAGTTCAATTGACCGACAGAAATTGTTATATGTGCACGAGACGCATGACCCG

>Km_sxtA_4

GTGAAGCGCCCATTCAAAATACGACTCGCTGAGAAATCGGATTTGTCCGAGCTTGTGAGACTAGAAGCTCTGACGTGGGATAAGCACCTGCAAGTACCCGAAAAGGTATTGAGCAGACGCTTGAAAACATCTCCTGCCACTGTATTGGTTTGTGAAGTTGACAAGAAGTTGGTTGGAGTCCTGTACATGCAGAAAATCGAGAGTCTGAGTTCAATTGACCGACAGAAATTGTTATATGTGCACGAGACGCATGACCCGGAAGGAAGTTTGATCCAGTTGATTTCAATCAACATCAACCCACAGTATGCTAGCA

>Km_sxtA_5

CTGGCAGACGGTTTCCGTCCCGAGGACACGGACAACCATGGTACTGGAGTTCTTATTCAGTACTCTGTCAACGATGTAATGGTAACTGATGCCAGCGAAACAAAGAGCTCGGCGACTAGCAAATCGAGTCAATCACTGCCAAATGGCACGCCTACCACGAAACAGATCTCTGCCTTTGACCAGATCAAGTCATTGATGGCAGAGTGGGGCTATACGGTGGACGACGAAGACTTGCACAGAGGTTTTTTCGATTATGGTATGGACTCTGTGGAGATTCAAGCCGTAAGAAACAAGCTGTCCGAC

>Km_sxtA_6

TGTCTGCCAGAGAGCTCATGCAAATGCAAGATGAGATTCGCAGCGTGTTGATGCAGCCAAGATATCAGGCGCGGTTTGCAGAAATTGCCAAAGAGTGCTATCCTGACTTCATCAAATACACTTATGAGATCGAACCCGTCCTCTGTGAAGTCGAAGGGCCTGTCATGCTACGCCATGGTTATATAGAGGAGAATACTCGTGCAGTTGTGCAAAAAAATCGGCCGTTGTTTTTTTCGCGAGTCTTCTTGAAGTATTGCCACAAGAACGATGAGGTCAGACGCCTTGGTCGGGAGATGTTGAGATTGACCGGCCAAGATCAAAGTTGGCCCTCAGAATGGTCGAGCACTATAGACGAGGCTGTCAATATTTTCGGTCGCATCATGTCAAGCAATTAGTTGGAAATTGATCATTTGTTCATGTAGAGCTCATAAACACCGTAACGCGAG

>Km_sxtA_7

GCTCCATCCGGTGCATTTCTCCGCAGCGGCCCAGAAAAATGTGCAACGAATTCCATCAGTAGCGTGCTGCACGTCCAACCATGGGCCAGCATGGGGGCGTTCGTGGCCAGACGAATGCCTTGCTCCAGAGCCTCGCGCAACGAAATCGGAGCATCCGAAGTATTCGATGGCACCGAAACCATCTCCTGCTCTGCCTCTAGATTCTCGTCCTCCTCCTGCACAGAGCCTAAGCGCCTCTGTTCCACGTACTGCGTAGTATCCGGAGCATAACCACGCATTGAATCCAAGTGATCGCTGAGACTGCTTACAGAAGGG

>Km_sxtA_8

GGTGTCAAAGCCTGGAATTCTCTGCGTGTAGAGAACACCAACAACCACACCATTGACTGTGCAAGCAAAGCATGTCGTGGGCGACGTAAGCAGGCGTTTCTTCAGGACCTCACTGGATGCCTGCATGTGTTTTTCCCACGCCAAAGCTTCAAGTTTGAGCAATGCGGGCATATCTGACAGCTCCGCGAGGCGAATCGAAAACGGACGCTTCATCAGATGCACGTTAACGATTCTGCAATAGCGTCCAAGCTCAGGGTAGCACTTGACAGTCTTGAAGCTCTTGGGAACAAGGCCCTCGTTTGCAGCAGCCATGGCGAATGCTGCCGGAGAGATTAGATGATGCCTCGCTAAGCTCTTTGTGATGTCATAGTGCAATGAAACGTTCTCGTTCATGTATCTTTTGGTCGTGGCAACGTTCAGCATCATCTCCTCCAACACACAGAGTCC

>Km_sxtA_9

GGAAGGAGGACTGGGTCTTCTGCCCCGCTTGCCAGAGGCGGCAGATTCTGACAACTCGTCGATGTTGTACTGAATCAACACGCCAGTCGCTCTGTTGTCCGTATCCTCGGGCCGATAGTTTGGCACTAATCTTTGTACAACTGCTCCGTAGCCCGTGTGAAAACCCAAGATGGGGTCAGTCAACTTGCCAGCAATATGTCTGTCCAAGTATGCCTGCATGCTGCCCTTGAAATTCTTGTAATCTCTGAAGAGCGTCACTGCACAAACGGAGCTGATTGAGACGTCCACTCGTGCCACGTATAGTGCAAATGCGCGCAACTCTGACCCAATGCCCATTGTCTT

>Km_sxtA_10

GTCCACTTTCGCTGACAGCTCATGCAGGACGTGCACAGAATCCAACGAATCCATCCCCAAGCCCGTGTCACCAAAGAAGGTGCTGTCCATCGTGTCGTCAGTAAGTTCATGACCGCACTTTTCTAAAAGTGGCTTCAAAAACGTGATGGTCTGCGTTGCCTTCTGCTCCACCGATTGGGAAGGAGGACTGGGTCTTCTGCCCCGCTTGCCAGAGGCGGCAGATTCTGACAACTCGTCGATGTTGTACTGAATCAACACGCCAGTCGCTCTGTTGTCCGTATCCTCGGGCCGATAGTTTGGCACTAATCTTTGTACAACTGCTCCGTAGCCCGTGTGAAAACCCAAGATGGGGTCAGTCAACTTGCCAGCAATATGTCTGTCCAAGTATGCCTGCATGCTGCCCTTGAAATTCTTGTAATCTCTGAAGAGCGTCACTGCACAAACGGAGCTGATTGAGACGTCC
